# Three new chondrosarcoma cell lines: one grade III conventional central chondrosarcoma and two dedifferentiated chondrosarcomas of bone

**DOI:** 10.1186/1471-2407-12-375

**Published:** 2012-08-28

**Authors:** Jolieke G van Oosterwijk, Danielle de Jong, Maayke AJH van Ruler, Pancras CW Hogendoorn, PD Sander Dijkstra, Carla SP van Rijswijk, Isidro Machado, Antonio Llombart-Bosch, Karoly Szuhai, Judith VMG Bovée

**Affiliations:** 1 Department of Pathology, Leiden University Medical Center, Albinusdreef 2, 2333, ZA, Leiden, The Netherlands; 2 Department of Medical Cell Biology, Leiden University Medical Center, Albinusdreef 2, 2333, ZA, Leiden, The Netherlands; 3 Department of Orthopedic surgery, Leiden University Medical Center, Albinusdreef 2, 2333, ZA, Leiden, The Netherlands; 4 Department of Radiology, Leiden University Medical Center, Albinusdreef 2, 2333, ZA, Leiden, The Netherlands; 5 Department of Pathology, University of Valencia Medical School, Avda, Blasco Ibañez 17, 46010, Valencia, Spain

**Keywords:** Bone neoplasm, Chondrosarcoma, Cell line, IDH1, IDH2, p16

## Abstract

**Background:**

Chondrosarcoma is the second most common primary sarcoma of bone. High-grade conventional chondrosarcoma and dedifferentiated chondrosarcoma have a poor outcome. In pre-clinical research aiming at the identification of novel treatment targets, the need for representative cell lines and model systems is high, but availability is scarce.

**Methods:**

We developed and characterized three cell lines, derived from conventional grade III chondrosarcoma (L835), and dedifferentiated chondrosarcoma (L2975 and L3252) of bone. Proliferation and migration were studied and we used COBRA-FISH and array-CGH for karyotyping and genotyping. Immunohistochemistry for p16 and p53 was performed as well as TP53 and IDH mutation analysis. Cells were injected into nude mice to establish their tumorigenic potential.

**Results:**

We show that the three cell lines have distinct migrative properties, L2975 had the highest migration rate and showed tumorigenic potential in mice. All cell lines showed chromosomal rearrangements with complex karyotypes and genotypic aberrations were conserved throughout late passaging of the cell lines. All cell lines showed loss of CDKN2A, while TP53 was wild type for exons 5–8. L835 has an IDH1 R132C mutation, L2975 an IDH2 R172W mutation and L3252 is IDH wild type.

**Conclusions:**

Based on the stable culturing properties of these cell lines and their genotypic profile resembling the original tumors, these cell lines should provide useful functional models to further characterize chondrosarcoma and to evaluate new treatment strategies.

## Background

Chondrosarcoma is a malignant bone neoplasm characterized by the deposition of a hyaline cartilaginous extracellular matrix. With an incidence of 1:50,000 it typically occurs in adults in their 3^rd^ to 6^th^ decade of life. Chondrosarcoma represents a heterogeneous group of tumors. Primary central chondrosarcoma is defined by the formation of hyaline cartilage with decreasing matrix production in higher grades and constitutes about 80% of all chondrosarcomas [1]. Dedifferentiated chondrosarcoma is characterized by a low-, or intermediate grade chondrosarcoma juxtaposed to a high grade anaplastic sarcoma and constitutes about 10% of all chondrosarcomas [2].

Both high grade conventional and dedifferentiated chondrosarcoma respond poorly to conventional chemo- and/or radiotherapy, have a high metastatic rate, and consequently have a very poor prognosis [3]. It is because of these features that there is an urgent need for model systems in pre-clinical research aimed at evaluating new targeted treatment strategies for chondrosarcoma [4].

Recently IDH1 and IDH2 mutations were found in conventional central and dedifferentiated chondrosarcomas [5]. IDH1 and IDH2 mutations are well known in gliomas [6], but are notoriously difficult to grow in culture [7]. This is a feature shared by, in particular, grade I chondrosarcomas. Recently, a new cell line derived from a grade II chondrosarcoma was published, CH-3573 [8]. Over the last years, cell lines derived from dedifferentiated chondrosarcomas have been developed [9,10]. In the pursuit of expanding the panel of cell lines we have succeeded in creating three new chondrosarcoma cell lines. L835 is derived from a grade III conventional chondrosarcoma, while L2975 and L3252 originate from dedifferentiated chondrosarcomas of bone. These three new cell lines provide a valuable addition to the current panel of chondrosarcoma cell lines.

## Methods

### Culture of human chondrosarcoma cells

Tumor-tissue derived from three resected specimens derived from one conventional and two dedifferentiated chondrosarcomas were used for culture. Samples were coded and all procedures were performed according to the ethical guidelines “Code for Proper Secondary Use of Human Tissue in The Netherlands 2002” (Dutch Federation of Medical Scientific Societies http://www.federa.org/sites/default/files/bijlagen/coreon/codepropersecondaryuseofhumantissue1_0.pdf). Specimens were washed 3x with RPMI1640 (Gibco, Invitrogen Life-Technologies, Scotland, UK) containing 1% penicillin/streptomycin (100U/mL), minced with razor blades and immersed in collagenase dispase overnight. After washing, the cells were transferred into small collagen-coated culture flasks and cultured in RPMI1640 supplemented with 20% heat inactivated Fetal Calf Serum (Gibco, Invitrogen Life-Technologies, Scotland, UK), 1% L-glutamax, and 1% penicillin/streptomycin (100U/mL). Cells were grown in a humidified incubator with 95% air and 5% CO_2_ and cultured until stably multiplying.

### COBRA-Fluoresence in-situ hybridization

COBRA-FISH on metaphase slides was performed as described previously [11]. For each cell line several cell culture passages were studied (L835: passage 17 and 35, L2975: passage 20 and 30, L3252: passage 7, 8, and 20) and karyotypes were described for each cell line according to the International System of Human Cytogenetic Nomenclature (ISCN) 2009.

### Expression of cartilaginous genes

RNA was isolated from L835 (passage 40), L2975 (passage 58), and L3252 (passage 21). Chondrogenic phenotype was assessed using RT-PCR for collagen I, IIB, III, and X, aggrecan, and SOX9 as described previously [12].

### Assessment of cell line identity

DNA isolation from cell pellets was performed using the wizard genomic DNA purification kit (Promega, Madison, WI) according to manufacturer’s instructions. DNA concentrations were measured using a Nanodrop ND-1000 spectrophotometer and quality was checked on a 1% agarose gel stained with ethidium bromide. Identity of cell lines was confirmed using the PowerPlex® 1.2 system (Promega Benelux BV, Leiden, The Netherlands). For L835 passage 36 was compared to primary tumor tissue, for L2975 passage 37 was used, and for L3252 passage 20 was compared to primary tumor tissue.

### Doubling time and migration assays

The RTCA xCelligence system (Roche Applied Sciences, Almere, the Netherlands), based on cell-electrode substract impedance detection technology [13], was used for doubling time and migration assays. Prior to starting experiments cell number curves were run to determine optimal growth curves and for doubling time experiments cell lines were plated at a density of 1,000 cells per well for L2975 and L3252 and 10,000 cells per well for L835 in growth medium (10% FCS in RPMI1640). For migration experiments, 100,000 cells were optimal.

For doubling time assays, 30 minutes after plating, view-plates were loaded into the RTCA station in the cell culture incubator. Cell index (CI) was acquired every hour.

Proliferation was monitored for 400 hrs. Every day plates were taken from the machine and most representative areas were photographed using a Zeiss axiovert 40C light microscope (Rijswijk, the Netherlands).

For migration assays, lower wells of the SIM plates (migration plates) were filled with growth medium (20% FCS in RPMI1640) as a chemoattractant, and cells were plated in the top wells in empty buffer (RPMI1640 only). CIM plates with 8 μm pores were loaded into the RTCA station in the cell culture incubator immediately after plating and cell index (CI) was acquired every 5 minutes. Migration was monitored for 24 hrs. Experiments were performed in triplicate.

### Mutation analysis

Mutation analysis was performed for TP53 (exons 5–8) [14], and IDH1 and −2 exons 4 [15] using direct sequencing of DNA as described (14;15). Mutation analysis for PIK3CA, KRAS, BRAF, EGFR was performed using hydrolysis probes assay [16] at L835 (passage 36), L2975 (passage 37), and L3252 (passage 20). Mutation analysis for TP53 was performed at those same passage numbers and IDH mutation analysis was performed at L835 passage 38 and 47, L2975 passage 31 and 46, and L3252 passage 20, as well as on DNA obtained from CH-3573 [8]. To determine expression of the IDH mutated allele cDNA was generated using 1 μg total RNA as described [12] for L835 (passage 38), L2975 (passage 31), and L3252 (passage 20). Primers were designed with primer3 software (http://frodo.wi.mit.edu/primer3/) and ordered from ISOGEN Bioscience BV (Maarssen, the Netherlands). PCR was done with the quantitative PCR core kit for SYBR green I supplemented with fluorescin (Eurogentec, Seraing, Belgium) on 0.2μL cDNA per reaction in an iCycler iQ Real-time Detection system (Bio-Rad Laboratories, Hercules, CA). PCR was done for 40 cycles. PCR products were purified using QIAquick PCR purification Kit (Qiagen, Hilden, Germany) according to manufacturer’s instructions. Purified products were sequenced by Macrogen (Amstelveen, the Netherlands) and resulting sequences were analyzed using MutationSurveyor DNA Variant Analysis software (Softgenetics, UK). Primer sequences and annealing temperatures are listed in Table 1.

**Table 1:** IDH primers

**Primer**			**Tm**	**Product size**
IDH1 genomic	Forward	CGGTCTTCAGAGAAGCCATT	59.4	113
IDH1 genomic	Reverse	GCCAACATGACTTACTTGATCC	58.6	
IDH2 genomic	Forward	AACATCCACGCCTAGTCC	56.3	90
IDH2 genomic	Reverse	CAGTGGATCCCCTCTCCAC	60.5	
IDH1 cDNA	Forward	CGGTCTTCAGAGAAGCCATT	59.4	131
IDH1 cDNA	Reverse	AGGCCCAGGAACAACAAAAT	56.4	
IDH2 cDNA	Forward	AGTGTGGCTGCAAGTGTGC	60.0	365
IDH2 cDNA	Reverse	GAGATGGACTCGTCGGTGTT	60.1	

### Array-CGH analysis

Array-CGH was performed on DNA derived from the primary tumor as well as from cultured cells of all three cases as described [17]. DNA of L835 passage 36, L2975 passage 37, and L3252 passage 20 was used. In brief, labeling of 1 μg DNA was performed using the BioPrime Total Genomic Labeling System (Invitrogen Corporation, Carlsbad, CA) following the manufacturer’s protocol. As reference, DNA from a commercial source (Promega Corporation, Madison, WI) was used. Labeled test and reference samples were mixed and hybridized as a gender mismatch. Hybridization was performed on an Agilent 105 k oligonucleotide array according to manufacturer’s instructions. Slides were scanned using the Agilent Scanner with 5 μm scan resolution. Scan images were processed with the Feature Extraction Software and the generated raw data files were analyzed using Genomic Workbench (Agilent Technologies, Santa Clara, CA). In short, the mean of the background corrected and Lowess normalized log2 ratios of identical features was calculated. Normalization of ratios was done using the overall values as well as the values of the control reporter probes on the array. Aberrations were calculated using the ADM-2 algorithm with a threshold of 8.6 and displayed with a moving average of 1 Mb.

### Tumorigenicity in mice

All the experimentation involving laboratory animals was approved by the Institutional Animal Care of Valencia University and the Local Government and was performed in accordance with the national legislation of Spain. Male nude mice were purchased from IFFA-CREDO (Lyon, France), and kept under specific pathogen-free conditions throughout the experiments. For each cell line, 2,000,000 cells were subcutaneously injected in a total of 3 mice (2 months old) under sterile conditions. Tumor was removed when size reached 4 mm in diameter and a fragment of non-necrotic tumor, about 3 to 5 mm^3^ in size, was used for xenografting into two new male nude mice. The second neoplasm was removed when the size reached 20 mm in diameter. From each tumor a part was snap-frozen in liquid nitrogen, a part fixed in formalin and embedded in paraffin, a part was used for xenografting into 2 new mice, and a part for further culturing of post-xenograft cell lines.

### (Immuno)histochemical analysis

L835 (passage 35), L2975 (passage 55), and L3252 (passage 17) cells were fixed in formalin and prepared using the Shandon Cytoblock cell block preparation system (Thermo Scientific, Etten-Leur, the Netherlands). Cells were embedded in paraffin according to standard laboratory procedures for tissue fixation. Sections (4-μm thick) of these paraffin blocks as well as from formalin fixed paraffin embedded original tumor tissue and xenograft passages, were used for H&E, toluidine blue, and immunohistochemistry for ki67, p53, and p16 according to standard procedures [18]. Details of the antibodies can be found in Table 2.

**Table 2:** Antibody properties

**Antibody**	**Clone**	**Dilution**	**Antigen retrieval**	**Blocking**	**Source**
**p16**	g175-405	1:800	Citrate	-	BD Pharmingen (550834)
**p53**	DO-7	1:800	Citrate	-	Dako (M7001)
**Ki67**	MIB-1	1:800	Citrate	-	Dako (M7240)

## Results

### Clinicopathological data

**Figure 1:**
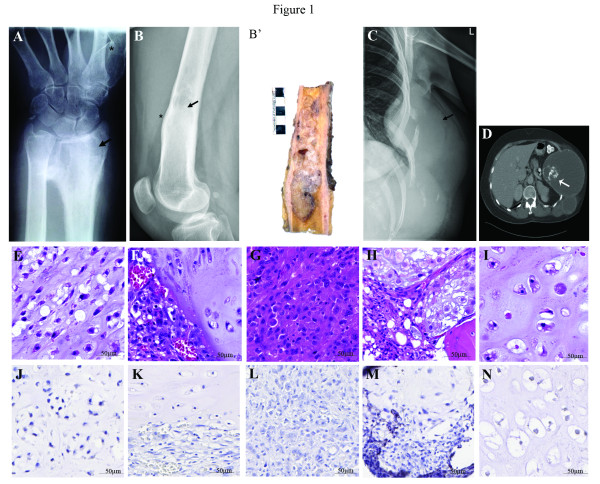
**Radiologic and histologic examination of original tumors.A**: L835 conventional radiography demonstrates a mildly expansile mixed lytic and sclerotic lesion in the distal radius with spiculated borders (arrow) and an irregular periosteal reaction. Note the presence of an enchondroma in the first metatarsal bone (asterisk). **B**: L2975 conventional radiography shows an ill-defined expansile mixed lytic and sclerotic lesion (arrow) originating from the distal femur diaphysis with endosteal scalloping of the anterior cortex (asterisk). The sclerotic areas within the lesion suggest osteoid matrix formation. **B’**: L2975 gross specimen shows intramedullar localization of dedifferentiated chondrosarcoma with chondrocytic and lobulated dedifferentiated compartments. **C**: L3252 conventional chest radiography shows a large lobulated mass originating from the left chest wall with extensive rib destruction. Subtle chondroid mineralization can be observed in the lesion. Radiological features are suggestive for a chondrosarcoma with a higher grade of malignancy. **D**: Axial CT image demonstrates chondroid mineralization (arrow) within the intra-abdominal component consistent with the diagnosis of chondrosarcoma. E: H&E staining of high grade L835 original tumor. H&E staining of L2975 original tumor shows anaplastic and cartilaginous component (**F**) and metastasis of the anaplastic component (**G**). H&E staining of L3252 original tumor shows anaplastic and cartilaginous component (**H**) and recurrence of the cartilaginous component (**I**). **J-N**: absence of p16 staining is observed in L835 original tumor, L2975 original tumor and metastasis, and L3252 original tumor and recurrence, respectively.

The tissue specimen of L835 was retrieved from the resection specimen of the local recurrence of a chondrosarcoma occurring in the left distal radius (Figure 1A) of a 54 year-old male. The primary tumor had been resected seven months earlier. Histological examination revealed a highly cellular cartilaginous neoplasm, with myxoid matrix changes and the presence of mitoses, and spindle cell changes at the edge of the lobuli, consistent with a primary central chondrosarcoma of bone, grade III according to Evans [19] (Figure 1E).

L2975 was derived from the resection of a metastasis of a dedifferentiated chondrosarcoma located at the spine of a 57 year-old male. The primary tumor was located at the right distal femur, and was originally resected eight months earlier (Figure 1B, B’). The primary tumor was 13.6 cm in size and histological examination revealed two components with a sharp interface indicative of dedifferentiated chondrosarcoma; a grade II chondrosarcoma was juxtaposed to a high grade anaplastic sarcoma (Figure 1F) with focal deposition of osteoid, indicating differentiation towards osteosarcoma. Histological examination of the spine metastasis revealed exclusively the high-grade anaplastic sarcoma, in which osteoid deposition was absent (Figure 1G).

L3252 is derived from the local recurrence of a chondrosarcoma located at the chest wall (costa) (Figure 1C, D) that had already metastasized to the spine and the lung at the time of first presentation. The primary tumor of this 52 year-old female demonstrated a cellular, partly myxoid and necrotic cartilaginous tumor, with mitoses, indicative of a grade III chondrosarcoma (Figure 1H). However, at local recurrence eight months later, areas of a cartilaginous tumor were intermingled with an anaplastic sarcoma lacking differentiation, indicating dedifferentiated chondrosarcoma. The tissue used for cell culture was derived from an area in which dedifferentiated areas were absent (Figure 1I).

### Establishment of cell lines and transmission light microscopy

**Figure 2:**
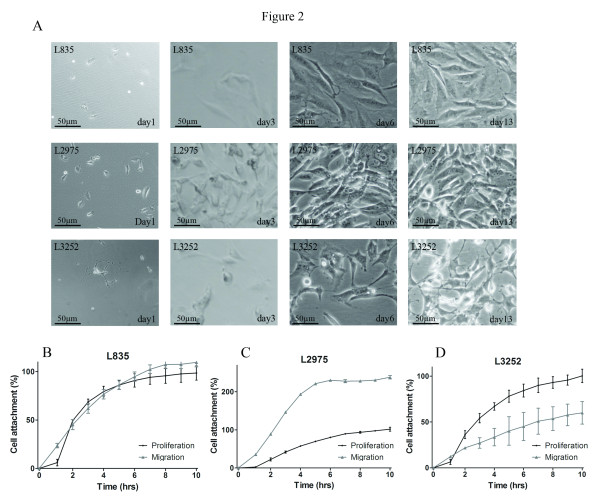
**Cell morphology and migration.A**: Light microscopy at 40x magnification. Top panel shows L835 cells with round nuclei do not fully populate the flask; day 13 is representative of a full flask for this cell line. Middle panel shows L2975 showing a full flask at day 6 already, multinucleated cells can be observed. Bottom panel shows L3252 still actively dividing, and dividing cells tend to detach and re-attach to the bottom of the flask. **B-D** Migration plotted against proliferation for the first 10 hours after plating. 1,000 – 10,000 cells were used in the proliferation assay and 100,000 cells in the migration assay. Though all cell lines show migrative capacity, L2975 cells are most successful and have high migrative activity during the first 4 hours after which the slope flattens.

L835 was passaged routinely in vitro for 50 generations, L2975 for 60 generations, and L3252 for 30 generations. The cell lines derived from dedifferentiated chondrosarcoma (L2975 and L3252) were noticeably easier to culture than the L835 cells. This was also reflected by Ki-67 staining on embedded cells, with proliferation rates of ~60% (L835) versus ~100% and ~80% for L2975 and L3252, respectively. The dedifferentiated chondrosarcoma cells were less susceptible to changes in culture conditions reflecting their more aggressive nature. Transmission light microscopy revealed L2975 cells to retain their elongated morphology when in confluence and L3252 cells to detach when reaching confluence (Figure 2A). Identity of cell lines was confirmed using the PowerPlex® 1.2 system (Promega Benelux BV, Leiden, The Netherlands). L835 passage 36, L2975 passage 37, and L3252 passage 20 showed identical STR loci when compared to their matching original tumors. Data are available on request. qPCR for expression of cartilage markers revealed expression of ColI, ColIII, aggrecan, and SOX9 in L835 cell line. L2975 cell line expressed ColI, ColIII, ColX, and SOX9. L3252B cell line expressed ColI, ColII, ColIII, ColX, aggrecan, and SOX9.

### Migration

All cell lines were able to migrate. Migration for L835 was at the same rate as cell attachment in the proliferation assay (Figure 2B). For L2975, cell migration occurred much more rapidly than cell attachment (Figure 2C). Moreover, due to high migration efficiency, and the fact that more cells were applied in the migration assay, the cell attachment percentage exceeds that of which is achieved in the proliferation assay. For L3252 cells, migration was observed but only for a small percentage of cells (Figure 2D).

### Transplantation into nude mice

**Figure 3:**
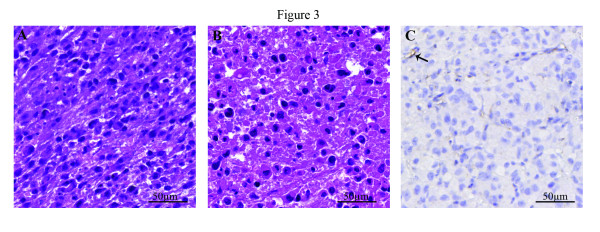
**Histologic examination of xenografted L2975.A**, **B**: H&E stainings of first and second passage of L2975 in nude mice showed L2975 cells had adapted a more rounded morphology. **C**: p16 staining of first passage confirmed absence of p16 expression; note positive vessel walls (arrow).

L2975 cells were subcutaneously transplanted into nude mice and a tumor of 4 mm in diameter was observed in 1 out of 3 mice after 3 months. A fragment of the resulting tumor was subsequently subcutaneously transplanted into 2 nude mice and a tumor of 20 mm in diameter was observed in 2 out of 2 mice. Morphologically, tumor cells of both the first and second xenograft showed a more epithelioid morphology (Figure 3A, B) while no deposition of cartilaginous matrix was observed at H&E or Toluidine Blue staining (results not shown). Immunohistochemistry showed that 90% of cells of both xenografts were positive for Ki-67 and were thus actively proliferating (results not shown). Cells obtained from both xenografts were successfully passaged. Xenografting of L835 and L3252 did not result in tumors in 8 months.

### COBRA-Fluorescence in-situ hybridization

**Figure 4:**
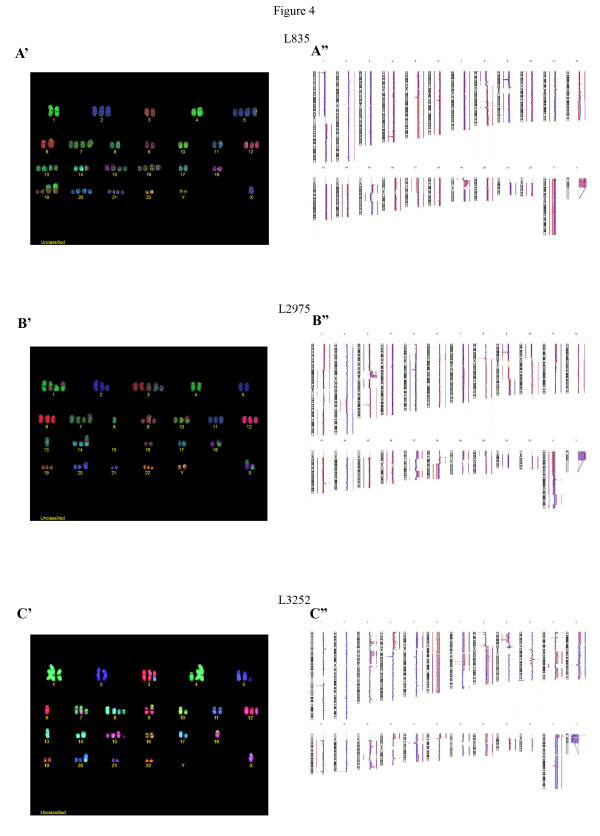
**COBRA-FISH and Array-CGH.A**: chromosomal analysis of L835 tumor and cell line showed numerical changes. **B**, **C**: chromosomal analysis of L2975, L3252 showed complex rearrangements consistent with the aggressive nature of dedifferentiated chondrosarcoma.

The cell line L835 showed a stable karyotype (Figure 4A’). There were many numerical changes with some translocations. The resulting karyotype at passage 35 was: 63-67 < 3n>,XY,-X,+Y,+der(1;19)t(1;19)(p11;p13.3)trp(19)(p13.3p13.2),der(1;19)t(1;19)(p11;p13.3)trp(19)(p13.3p13.2),-3,-4,+5,-6,+7,-8,-9,-10,-11,+13,t(14;15)(q22;q24),+der(15)t(14;15)(q22;q24)del(15)(q21q24),+16,-17,i(17)(q10),-18,-19,+20,+21,-22.

L2975 also revealed a stable karyotype at passage 30 with many numerical changes and complex rearrangements (Figure 4B’). The resulting karyotype was: 61-65 < 3n > der(X;4)(p10;p10),der(X;8)(q10;q10),-Y,+der(Y;9)(p10;p10)*x*2,+der(1;15)(q10;q10),der(1;1)(q10;q10)t(1;6)(q21;q24)t(1;8)(q31;p11),der(1;8)(p10;p10),del(2)(q22q32),-4,-5,+der(7;15)(q10;q10),t(9;16)(q10;q10)*x*2,der(10;18)(q10;q10),-13,der(13;13)(q10;q10),der(14;15)(q10;q10)t(8;15)(q11;q21),-15,-15,-16,-17,i(17)(q10),-18,der(18)t(1;18)(p31;q22),der(20)t(X;20)(q23,p12),-21,+mar3x.

L3252 was stable at passage 20 with many numerical changes and complex rearrangements (Figure 4C’): 51 < 2n+>,X,-X,+der(3;11)t(3;11)(p10;q10)t(11;17) (q14;q22), der(4;8)t(4;8)(q10;p10),der(4),+der(5),der(6;15)t(6;15)(p10;q10), der(7)t(7;18),der(7),+der(8)*x*2,der(8),der(9),der (10),+12,der(13;13)(q10;q10),-13,der(17),i(17)(p10;p10)der(18)t(7;18),+der(20), der(21),+mar.

### Array-CGH and mutation analyses

In L835 and L2975, all aberrations present in the tumor were retained in the cell lines. L835 showed a homozygous CDKN2A deletion in both the original tumor and the cell line (Figure 4A”). L2975 and L3252 both showed a homozygous deletion in the cell line (Figure 4B” and 4C”), the deletion status of the primary tumor was difficult to assess due to low tumor content and consequently resulting suppressed ratio profiles. For both L835 and L2975 clear DNA copy number alterations could be observed in the original tumor samples that were enhanced in the cell lines. For L3252 tumor DNA this was less pronounced. On close examination of the profile, however, one can observe small copy number changes consistent with those observed in the cell line. We previously demonstrated L835 (passage 38) to harbor an IDH1 R132C mutation and L2975 (passage 31) an IDH2 R172W mutation [15]. We here show that L3252 (passage 20) is wild type and that also later passages of L835 (passage 47) and L2975 (passage 46) retain the IDH mutation. Using cDNA we found the mutated IDH alleles to be expressed (results not shown). No hotspot mutations were detected for TP53, PIK3CA, BRAF, KRAS, and EGFR in any of the cell lines.

### Immunohistochemical characterization

Since p16 is frequently silenced in human chondrosarcoma [18] we evaluated p16 in the original tumors, the cell lines and the L2975 xenografts using immunohistochemistry. All primary tumors (Figure 1J, L, M) and recurrences (Figure 1L, N) were negative for p16. Consistent with the tumor tissue, all derived cell lines lacked p16 protein expression (Table 3), as did xenografts created from L2975 (Figure 3C). We proceeded to test embedded cells from all cell lines shown in Table 3 for p16 expression and found all to be negative. Immunohistochemistry showed nuclear expression of p53 in 10-20% of the tumor cells in L835 and L3252 primary tumor and cell line, while L2975 tumor, cell line, and xenograft were completely negative (results not shown).

**Table 3:** Overview of chondrosarcoma cell lines and their characteristics

**Cell line**	**Tumor subtype**	**Histological grade**	**Passage**	**IDH1**^**1**^	**IDH2**^**1**^	**p53**^**2**^	**p16**^**3**^	**Reference**
**Conventional Chondrosarcoma**								
SW1353	Solitary Central	II	p50	wt	R172S	V203L	-	ATCC
JJ012	Solitary Central	II	P26	R132G	wt	G199V	-	(30)
CH3573	Solitary Central	II	P60	wt	wt	T201-	na	(8)
CH2879	Solitary Central	III	P31	G105G	wt	wt	-	(31)
OUMS27	Solitary Central	III	P29	wt	wt	wt	-	(32)
L835	Solitary Central	III	p51	R132C	wt	wt	-	Present Study
**Dedifferentiated Chondrosarcoma**								
L2975	Dedifferentiated		p59	wt	R172W	wt	-	Present Study
NDCS1	Dedifferentiated		P22	wt	wt	C242S	-	(9)
L3252	Dedifferentiated		P26	wt	wt	wt	-	Present Study

^1^IDH mutations for existing cell lines were described in (15).

^2^p53 mutations for existing cell lines were described in (33).

^3^p16 mutations for existing cell lines were described in (18).

na: not available.

## Discussion

Chondrosarcoma is the second most common primary sarcoma of bone and to date unresectable chondrosarcomas have a poor outcome [3]. Grade III and dedifferentiated chondrosarcomas are extremely aggressive in nature and there is an urgent need for model systems facilitating research in order to develop novel therapeutic strategies. Growing chondrosarcoma cells in culture, however, is a challenge and well growing chondrosarcoma cell lines are sparse. We present here the establishment and characterization of three new chondrosarcoma cell lines originating from grade III and dedifferentiated chondrosarcoma.

Recently chondrosarcoma has been found to harbor IDH1 and IDH2 mutations (5;15) and we published that the mutation is retained in a subset of chondrosarcoma cell lines [15]. In glioma IDH mutations seem to be the earliest event in gliomagenesis even before TP53 mutations occur [20]. In conventional chondrosarcoma we observe a similar phenomenon, where IDH mutations are present already in a high percentage of low-grade tumors and TP53 mutations are observed to increase with grade (4;5;15). Cell lines created from IDH mutant gliomas have been reported to eliminate their IDH mutation under standard culture conditions [7]. Recently, however, a glioma cell line carrying an endogenous IDH1 R132H mutation was published, but this cell line showed a slow growth rate in culture [21]. We here present three chondrosarcoma cell lines, one carrying an IDH1 R132C mutation, one carrying an IDH2 R172W mutation, and one wild type for IDH mutations with stable karyotypes and steady growth patterns. These cell lines show numerical changes and additional mutations. We speculate that in IDH mutant chondrosarcoma the acquisition of additional mutations as we have shown here have facilitated their growth in culture.

The inactivation of tumor suppressor genes is a well-known phenomenon in cancer and p16 mutations have been reported in 20-41% of human chondrosarcomas [22-25]. Interestingly, all studies observed loss of p16 to be correlated with increasing histological grade in conventional chondrosarcoma. Recently, we showed inactivation of p16 in 30/38 (79%) dedifferentiated chondrosarcoma cases [26]. We previously published three chondrosarcoma cell lines to be negative for p16 using western blot [18] and upon overexpression of p16 using lentiviral vectors the metabolic activity and cell viability of these cell lines was decreased, indicating loss of p16 to play a role in the proliferative capacity of chondrosarcoma cells. Introduction of p16 in the endogenously TP53 mutant HT-1080 fibrosarcoma cell line, which was recently reported to carry an IDH1 R132C mutation [5], also led to cell cycle arrest and growth inhibition [27-29]. We report here three new chondrosarcoma cell lines lacking p16 expression based on a homozygous deletion of the CDKN2A locus as shown by aCGH analysis, and confirmed loss of p16 expression using immunohistochemistry. Moreover, aCGH analysis showed a copy number loss around the 17p13.1 locus in L835, whereas a copy number gain was observed in L2975 and L3252. However, mutation analysis for TP53 showed no activating mutations in exons 5–8, and immunohistochemistry showed no p53 overexpression. Together, our data suggest that while IDH mutations are important as early events in a subset of chondrosarcomas, additional inactivation of p16 may be crucial for acquiring a more aggressive phenotype.

The literature presents us with 5 conventional chondrosarcoma cell lines that have been well characterized using pathological, immunohistochemical, and molecular genetic methods [8,30-32], and we here present L835 as an additional cell line. We previously published L835 to be able to form 3D pellets [33] and we now show it to be highly stable in culture. L835 cell line showed a slower growth rate compared to the dedifferentiated chondrosarcoma cell lines. In all cases complex genome alterations were observed. Dedifferentiated chondrosarcoma is comprised of two separate components, a high grade anaplastic component and a low to intermediate grade cartilaginous component [2]. The histogenesis has been under debate but evidence points to a single precursor cell with early separation of the two components as a small number of genetic changes is identical in both components, with additional genetic alterations in the anaplastic component [26,34]. Indeed, 3 out of 3 dedifferentiated chondrosarcomas with IDH1 mutations carried the mutation in both components [26]. Moreover, 79% of the anaplastic and 82% of the cartilaginous components show loss of p16 expression [26]. L2975 and L3252 were both derived from the recurrence of a dedifferentiated chondrosarcoma; both cell lines exhibited a higher growth rate in vitro, than the L835 cell line, but the cells in culture expressed chondrogenic markers. L2975 proved to be the most aggressive cell line both in culture and in our in vitro migration assay, which may explain why this was the only cell line to be successfully xenografted. We show here the use of L2975 dedifferentiated chondrosarcoma cells with an IDH2 R172W mutation in mouse models, which can be an important asset in the research for new treatment strategies.

## Conclusions

We report the establishment and molecular, genetic and functional characterization of one grade III (L835) and two dedifferentiated chondrosarcoma (L2975 and L3252) cell lines. This represents a substantial addition to the already existing panel of chondrosarcoma cell lines, which together may reflect their heterogeneity. In addition to the existing cell lines these cell lines present the field with an extensive model system as heterogeneous in IDH1 and IDH2 and TP53 mutations as the tumors they are derived from. This panel can be implemented in studies ascertaining human chondrosarcoma tumorigenesis, should provide useful tools in the ongoing search for new targeted therapies, and aid in expanding our knowledge on the role of IDH1 and IDH2 mutations in chondrosarcoma formation.

## Competing interests

The authors have no conflict of interest to declare.

## Authors’ contributions

JGvO, JVMGB, KS, PCWH, and AL-B conceived of the study. JGvO, DdJ, MAJHvR, IM, and KS performed the experiments. JGvO, JVMG, PDSD, and CSvR participated in analysis and interpretation of patient data and all authors read and approved the final manuscript.

## Financial support

Netherlands Organization for Scientific Research (917-76-315: J.G.v.O, and J.V.M.G.B.), Dutch Cancer Society (UL2010-4873: J.G.v.O. and J.V.M.G.B.). Leiden University Medical Centre and University of Valencia Medical School are partners in the context of EuroBoNet, a European Commission granted Network of Excellence to study the pathology and genetics of bone tumors (018814).

## Pre-publication history

The pre-publication history for this paper can be accessed here:

http://www.biomedcentral.com/1471-2407/12/375/prepub
